# Beyond One-Size-Fits-All: Addressing Methodological Constraints in Novel Antimicrobials Discovery

**DOI:** 10.3390/antibiotics14080848

**Published:** 2025-08-21

**Authors:** Silvia Puxeddu, Serena Canton, Alessandra Scano, Ilenia Delogu, Andrea Pibiri, Cristiana Cabriolu, Sarah Vascellari, Francesca Pettinau, Tiziana Pivetta, Guido Ennas, Aldo Manzin, Fabrizio Angius

**Affiliations:** 1 Department of Biomedical Sciences, Section of Microbiology and Virology, University of Cagliari, 09042 Cagliari, Italy; 2 Department of Chemical and Geological Sciences, University of Cagliari, 09042 Cagliari, Italy; 3 Research Unit of the National Consortium of Materials Science and Technology (INSTM), University of Cagliari, 09042 Cagliari, Italy; 4 Institute of Translational Pharmacology, National Research Council, 09010 Pula, Italy

**Keywords:** Kirby-Bauer, disk diffusion, broth dilution, agar dilution, MIC, MBC, antimicrobial susceptibility testing

## Abstract

**Background**: Antimicrobial resistance is a growing global health concern that requires multiple strategies to be tackled effectively. While the discovery of new antimicrobial molecules is essential, the repurposing of existing compounds also plays a significant role. Standard methods to evaluate antimicrobial efficacy, regulated by the Committee on Antimicrobial Susceptibility Testing (EUCAST) and the Clinical and Laboratory Standards Institute (CLSI), such as the determination of minimum inhibitory concentration (MIC) and minimum bactericidal concentration (MBC), are available. However, several potential antimicrobics show interference with these standard methods, resulting in underestimated activity and their premature dismissal from further studies. This work compares reference methods in evaluating different compounds with unique physico-chemical characteristics. We aim to demonstrate that combining different susceptibility tests is mandatory for a successful preclinical screening of antimicrobial compounds. **Methods**: A selection of substances including natural extracts, both free and in the form of nanocomposites with fumed silica, ionic liquids, ozonated oils, commercial and pure antibiotics, was tested using broth microdilution, disk diffusion, and agar dilution. These methods were chosen following EUCAST and CLSI guidelines, and comparisons were made to evaluate their applicability and limitations for non-conventional substances. **Results**: The study highlighted significant variability in the outcomes depending on the method used, especially for substances with intrinsic properties such as high viscosity, poor solubility, or specific interactions with the testing medium. In several cases, the use of a single standard method failed to accurately reflect the real antimicrobial activity, leading to potential misinterpretation of effectiveness. **Conclusions**: A combined methodological approach is recommended to overcome the limitations of individual techniques. The integration of multiple reference methods offers a more accurate screening strategy for identifying and characterizing new and repurposed antimicrobials.

## 1. Introduction

Antimicrobial resistance (AMR) is an increasingly urgent global issue. It demands the evaluation of innovative strategies aimed at discovering new molecules capable of counteracting known resistance mechanisms and preventing the emergence of new ones [1]. It is well established that every antibiotic used in clinical practice selects for bacteria owning intrinsic or acquired resistance mechanisms, and thus has a limited period of efficacy [2]. AMR occurs when antibiotics are used inappropriately, such as in cases of incorrect prescriptions, poor adherence to therapy, or widespread use in agriculture and livestock farming [3]. At this rate, if new effective substances are not discovered or synthesized, we may face a concerning return to a pre-antibiotic era. According to data from the Institute for Health Metrics and Evaluation, antibiotic resistance could lead to 10 million deaths annually, becoming one of the leading causes of death worldwide [4]. Under this scenario, innovative pharmacological approaches are being developed to enhance the efficacy of existing antibiotics [5,6]. Among these, nanocarrier-based delivery systems—particularly lipid vesicles and functionalized chemical vectors—are of remarkable interest, as they enable controlled and targeted drug release [7,8,9]. These advanced technologies improve the bioavailability, stability, and penetration of antibiotics at the site of infection, proving especially effective against infections by multidrug-resistant strains and in the disruption of bacterial biofilms [10,11].

In parallel, research is also increasingly focusing on antimicrobial products of natural origin [12]. Phytochemicals like alkaloids, tannins, terpenoids, essential oils, flavonoids, polyphenols, lectins, secondary metabolites, and many other organic constituents cooperatively act against microorganisms [13,14,15,16,17,18]. Despite a large number of scientific publications reporting on the use of natural products for treating microbial infections, they suffer from a lack of stability [19,20,21,22]. Physical and chemical degradation of the natural compounds can occur due to temperature, light or pH, which could be avoided by incorporating them into delivery systems [7,8,9,23]. Such strategies can also increase their solubility in the body fluids, hence giving rise to enhanced bioavailability and pharmacological activity [24].

Other options have been recently offered using ionic liquids, compounds consisting of organic cations and inorganic or organic anions. Among several features such as non-volatility, non-flammability, low melting points, high thermal stability and adjustable physico-chemical properties, ionic liquids also show antimicrobial activity [25,26,27,28]. Finally, it has been recently reported that ozonated oils may represent a promising approach as antimicrobial agents [29]. Their antimicrobial activity comes from different types of peroxide species and aldehydes, also known as ozonides, developed during the ozonation process of refined vegetable oils. Ozone, in fact, reacts with the carbon–carbon double bonds of the unsaturated fatty acids of the oils, giving rise to the formation of active species [29].

A broad host of opportunities seems to be offered by these new classes of antimicrobials. Hence, the question is whether we are prepared to test their antimicrobial activities. Are current evaluation techniques still adequate, or is there a need to develop more specific and sensitive approaches? Standard evaluation methodologies are generally effective for traditional antimicrobials like antibiotics or disinfectants, but there may be some challenges when used to test these unconventional substances. Their complex composition, solubility, or volatility can make it more difficult to apply traditional testing methods. Moreover, to evaluate the susceptibility of classical antimicrobial molecules, it is essential to follow standardized protocols that ensure reproducibility and reliability of the results, in particular in clinical settings. For this purpose, two internationally recognized reference bodies, the European Committee on Antimicrobial Susceptibility Testing (EUCAST) and the Clinical and Laboratory Standards Institute (CLSI), developed detailed guidelines for antimicrobial susceptibility and determination of minimum inhibitory concentration (MIC) and minimum bactericidal concentration (MBC) of antimicrobial agents. MIC is defined as the lowest concentration of an antimicrobial agent that visibly inhibits the growth of a microorganism after a standard incubation period, generally 16–20 h for fast-growing bacteria [30,31]. MBC, on the other hand, refers to the lowest concentration of the antimicrobial agent capable of killing at least 99.9% of the initial bacterial inoculum [32]. These parameters are fundamental for distinguishing between bacteriostatic and bactericidal effects and for guiding appropriate therapeutic decisions [30,32]. EUCAST provides recommendations for MIC determination using broth microdilution in accordance with ISO 20776-1 standards [33]. Its guidelines also include the definition of clinical breakpoints and epidemiological cut-off values (ECOFFs), which are essential for the interpretation of antimicrobial susceptibility test results [34,35,36]. In parallel, CLSI has developed document M07 that describes broth dilution methods (macro- and microdilution) and agar dilution for MIC determination, as well as document M26-A for MBC determination [30,32]. The adoption of these standards allows for data comparability among laboratories, traceability of results, and a consistent approach in evaluating the antimicrobial potency of new compounds.

**Figure 1:**
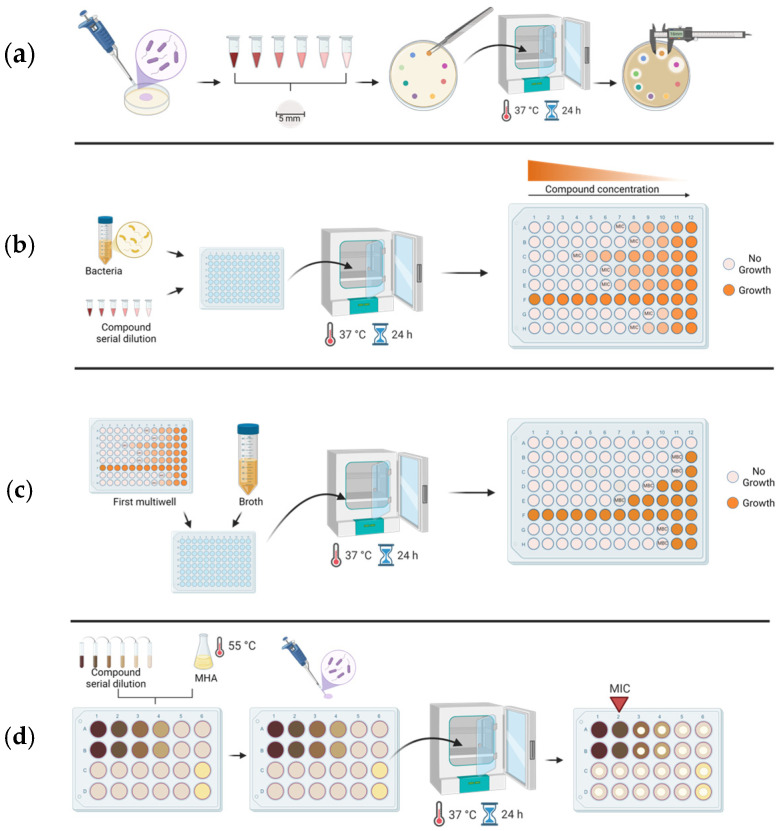
Schematic representation of the antimicrobial susceptibility testing methods employed in this study. (**a**) Disk diffusion assay (Kirby–Bauer method). Inhibition zones are measured to assess antimicrobial activity. (**b**) Broth microdilution for MIC detection, which is defined as the lowest concentration that does not significantly differ from that of the original inoculum by turbidimetry assay. (**c**) Broth microdilution for MBC detection, defined as the lowest concentration that does not significantly differ from that of the original inoculum after 24 h incubation at 37 °C by turbidimetry assay. (**d**) Agar dilution for MIC detection, defined as the lowest concentration showing no visible bacterial colonies. For details, see Section 4.

Antimicrobial susceptibility testing (AST) can be performed using various methodologies such as disk diffusion, which measures the inhibition zone produced by antibiotic-impregnated disks on agar plates; broth microdilution, involving serial dilutions of compounds in liquid culture to determine MICs; and agar dilution, where varying concentrations of antimicrobial agents are incorporated into agar to assess bacterial growth inhibition directly on solid media (Figure 1).

According to the EUCAST [36] and CLSI [37] guidelines, these methods are considered alternative approaches, and their applicability may vary depending on the specific antimicrobial compound under investigation. For certain molecules, such as colistin or glycopeptides, the use of specific techniques (e.g., broth microdilution) is strongly recommended due to the limitations in accuracy and reproducibility associated with other methods [36,37]. Several studies have already encountered this issue and have focused on identifying the most suitable testing technique based on the nature of the compound [38,39,40]. This approach became necessary as it was progressively recognized that many compounds, originally marketed for other purposes (e.g., anti-inflammatory, anti-edematous), have revealed antimicrobial effects that were previously undetected due to the inadequacy of conventional testing methods to account for their physico-chemical characteristics [41,42,43]. This underscores the importance of selecting the most appropriate testing method based on the characteristics of each antimicrobial agent. Consequently, the use of a single standardized technique across all compounds in preclinical research may lead to inaccurate conclusions or to a failure in detecting antimicrobial activity, especially when the selected method is suboptimal for certain active ingredients.

With the present study, we aim to take a further step forward by comparing different AST techniques applied to substances of diverse nature. We would like to highlight how reliance on a single method may lead to misleading conclusions regarding antimicrobial efficacy. To this end, we propose a more comprehensive evaluation strategy that can accurately reflect the full activity spectrum of a substance, regardless of its chemical nature, ensuring that the results are reliable and representative. This could pave the way for the reassessment of numerous compounds already in use, thereby expanding the arsenal of available antimicrobial agents without the need for investment in the development of new molecules [41,42,43].

## 2. Results

A selection of substances was tested using broth microdilution, disk diffusion, and agar dilution for antimicrobial activity determination against *S. aureus* and *E. coli* as experimental models for Gram-positive and -negative bacteria (Table 1). The substances tested are labeled in the body of text and in the tables with full names and acronyms.

**Figure 2:**
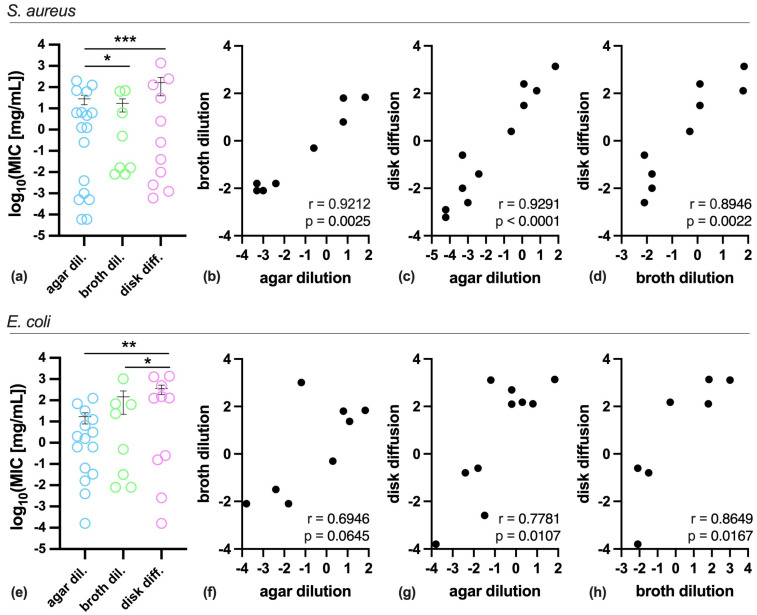
MIC comparison and correlation between the antimicrobial testing methods. Data are reported as scatter plot of individual values and mean ± SEM on a log scale for *S. aureus* (**a**) and *E. coli* (**e**), with the statistical significance indicated as * *p* < 0.05, ** *p* < 0.01, and *** *p* < 0.001, as calculated by the Wilcoxon matched-pairs signed rank test. (**b**–**d**,**f**–**h**) MIC correlation between the antimicrobial testing methods. Spearman’s r coefficient and statistical *p* value are indicated in each graph as calculated by correlation analysis. Data are significant for *p* < 0.05.

As shown in Figure 2, the basic comparative analysis of data revealed that MIC values obtained by disk diffusion and broth dilution methods were significantly different compared to those obtained by agar dilution, when tested against *S. aureus* (Figure 2a), whereas agar and broth dilution methods showed similar results when tested against *E. coli*, though disk diffusion was the only significantly different when compared to the other methods (Figure 2e). However, these differences are likely underestimated as, among a total of 18 compounds, we obtained 17, 8 and 11 MIC values against *S. aureus* by agar dilution, broth dilution and disk diffusion, respectively, and 15, 8, and 10 against *E. coli* (Table 1). In addition, non-parametric Spearman correlation analysis revealed significant positive correlations for all pairs analyzed for *S. aureus* (Figure 2b–d), while for *E. coli* only disk diffusion showed a significant positive correlation with agar and broth dilution methods (Figure 2g,h).

**Figure 3:**
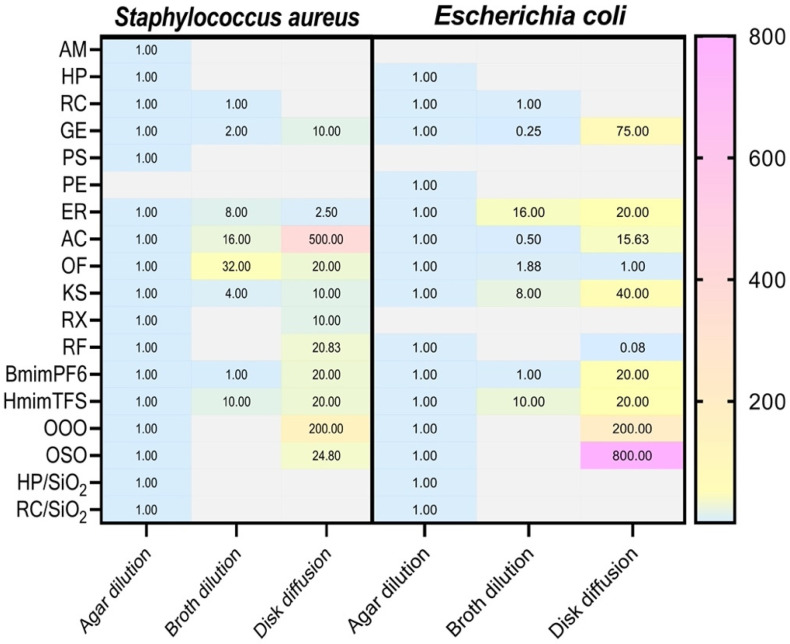
Graphical representation of antimicrobial activity data compared to agar dilution. Individual MIC values of the tested compounds obtained by disk diffusion, agar dilution, and broth dilution methods, against *S. aureus* and *E. coli*, are reported as fold change differences calculated as ratios vs. the agar dilution results and are represented by a color gradient scale (from light blue to magenta). The individual values are reported within the graph. Light gray empty boxes indicate not assessable data. AM (*Arnica montana*), HP (*Harpagophytum procumbens*), RC (*Rosa canina*), GE (grapefruit extract), PS (polyphenol Solgar), PE (polyphenol Emma), ER (erythromycin), AC (amoxicillin/clavulanic acid), OF (ofloxacin), KS (kanamycin sulfate), RX (rifaximin), RF (rifampicin), OOO (ozonated olive oil), OSO (ozonated sunflower oil), HP/SiO_2_ (*Harpagophytum procumbens*/SiO_2_), RC/SiO_2_ (*Rosa canina*/SiO_2_).

It is noteworthy that in some cases, even when a correlation is present, the MIC values between methods may be very different. This is the case of ozonated olive oil (OOO) and amoxicillin/clavulanic acid (AC) against *S. aureus*, which reach MIC values by disk diffusion 200 and 500 times higher compared to those obtained by agar dilution, respectively. The same was observed for ozonated sunflower oil (OSO) and OOO against *E. coli* with values 200 and 800 times higher (Figure 3). Overall, these results indicate substantial variability in antimicrobial efficacy parameters depending on the sample tested, bacterial species, and most importantly, the testing method (Table 1, Figure 2 and Figure 3).

Concerning natural extracts, *Arnica montana* (AM) did not show activity against either the Gram-positive or -negative bacteria tested with any of the methods used. On the contrary, *Harpagophytum procumbens* (HP) and *Rosa canina L*. (RC) by disk diffusion assays indicated no apparent antimicrobial activity with inhibition halos absent even at the highest concentration tested (200 mg/mL), while agar and broth dilution methods revealed measurable MIC and MBC values, in some cases below 12.5 mg/mL (Table 1 and Table 2). The same trend was observed for polyphenols (PS for Solgar and PE for EMMA) and for grapefruit extract (GE), the latter when tested against *E. coli* (Table 1 and Table 2).

Similar behavior was observed when testing ionic liquids. 1-Butyl-3-methylimidazolium hexa-fluorophosphate (BmimPF_6_) showed an MIC value of 1380 mg/mL when tested by the disk diffusion method, while the value decreased to 69 mg/mL using agar or broth dilution methods. A decreased MIC value was also observed for 1-Decyl-3-methyl imidazolium bis (trifluoromethylsulfonyl)imide (HmimTFS) when comparing the results obtained by disk diffusion and dilution methods. The different MIC values obtained for the two ionic liquids featuring the same cation in the structure can be ascribed to the different length of the alkyl chain. Several studies reported that the length of the alkyl chain linked to the cation plays an important role in the ionic liquids’ antimicrobial activity [25,26,27,28]. The aforementioned discrepancies underscore the limitation of diffusion-based assays due to a steric hindrance in the solid agar structure, so that it results in a diffusion block for the tested substances, as recently reported in the literature [25,44] and also experienced in our previous studies [45]. Limitations of this method were also reported in the literature when using it for detecting the activity of poorly soluble molecules [46,47]. This does not mean that the substances have no antimicrobial activity, but it underlines the difficulty of leaching and acting against the bacteria. Molecular weight, solubility, and diffusion rate could, in fact, impact the diffusion of test substances in the agar [44].

Moreover, we observed that several potent bactericidal agents, including rifampicin (RF) and rifaximin (RX), exhibited significantly higher MIC values by disk diffusion compared to broth and agar dilution (over 20-fold higher for RF against *S. aureus*) (Table 1 and Figure 3). These differences stem from diffusion-based assay limitations, where hydrophobicity, high molecular weight, and poor solubility hinder compound penetration through agar, leading to underestimated antimicrobial activity. Likewise, synthetic antibiotics, such as ofloxacin (OF) and kanamycin sulfate (KS), showed variability among the three methods, each presenting strengths and limitations depending on the specific molecules tested and the microorganisms considered [48]. This confirms the necessity to test synthetic molecules by all three methods. Indeed, as reported in Table 1, some antibiotics exhibited MIC values that varied by several orders of magnitude depending on the method used such as for AC ranging from 0.25 to 0.0005 mg/mL, while others (e.g., OF) showed consistent results across all methods. This suggests that medium composition and nutrient content may influence drug availability or bacterial growth dynamics [35,49,50].

Notably, natural extract/silica nanocomposites (HP/SiO_2_, RC/SiO_2_) showed no activity when assessed by broth dilution. In the case of the nanocomposites, the optical density measuring could be affected by the silica optical properties, such as light transmission and reflection [51]. 

Moreover, as shown in Table 1, remarkable differences are observed in MIC values obtained by agar-based assays. Indeed, when comparing data obtained by agar diffusion and agar dilution methods, for HP/SiO_2_ vs. *E. coli*, the MIC value decreased from >125 to 47.25 ± 21.57 mg/mL as well as from >125 to 93.75 ± 44.19 mg/mL for RC/SiO_2_ vs. *S. aureus*. This suggests higher bioavailability of the active ingredients and thus, an increased possibility to achieve a bacteria–drug interaction when using the agar dilution test [46]. This also aligns with studies showing hydrophobic compounds in liquid media adsorb onto solid surfaces, as for example, the diffusion disk, making MICs higher [52]. Differential diffusion of the extract components in the aqueous media could also influence the test result, so that the different chemicals from the extract may diffuse at different rates, resulting in uneven distribution of antimicrobial compounds around the disk [53].

When evaluating the MIC by broth microdilution, it is also common practice to assess the MBC to distinguish the compound’s activity (bacteriostatic vs. bactericidal). In some cases, the MBC may be detectable even when the MIC is not [29], which can be attributed to the nature of the compound—such as interference with spectrophotometric readings or precipitation. In many other instances, the absence of a detectable MIC leads to the MBC being omitted from the analysis. This potentially results in the loss of valuable information regarding the compound’s full antimicrobial spectrum against that organism, an oversight that may lead to incorrect conclusions (Table 1 and Table 2).

## 3. Discussion

**Figure 4:**
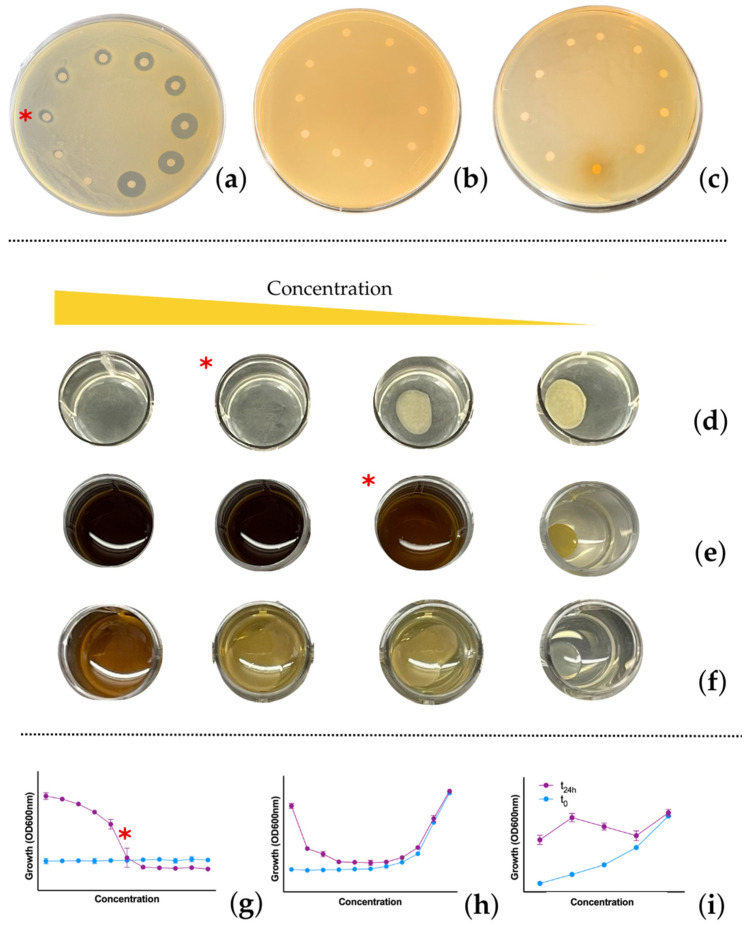
Representative images of antimicrobial susceptibility testing outcomes. (**a**–**c**) Disk diffusion method showing when (**a**) MIC is identified or not (**b**,**c**), with some diffusion of extract components in the agar. (**c**–**f**) Agar dilution method when (**d**,**e**) MIC is determined; the presence or absence of bacterial growth could be clearly determined (**e**) even with darkly colored substances. (**f**) Growth is evident at all concentrations tested; hence, MIC determination is not possible. (**g**–**i**) Broth dilution method. (**g**) MIC was correctly identified. This was not the case in (**h**), and (**i**) where the substance under investigation interfered with spectrophotometric readings, preventing accurate detection of the bacterial signal at higher concentrations. MIC values are indicated by a red asterisk. (**a**) rifaximin on *S. aureus*, (**b**) *Arnica montana* extract on *E. coli*, (**c**) *Harpagophytum procumbens* on *E. coli*, (**d**) erythromycin on *S. aureus*, (**e**) *Harpagophytum procumbens* on *S. aureus*, (**f**) *Arnica montana* extract on *E. coli*, (**g**) amoxicillin/clavulanic acid on *E. coli*, (**h**) rifaximin on *E. coli*, (**i**) *Arnica montana* extract on *E. coli*.

In this study we showed how the combination of different reference methods provides a meticulous approach for the identification and characterization of potential antimicrobial agents with distinct intrinsic properties. Although the antimicrobial activity of most of the tested substances was detected using the agar dilution method (Figure 3 and Figure 4), this technique has critical limitations. One major constraint is that the test molecules must remain chemically and structurally stable when incorporated into agar at 55 °C [53]. Additionally, this method is technically demanding, requiring rapid execution and a high level of precision from the operator dispensing molten agar [54]. Furthermore, agar dilution is a qualitative rather than quantitative method. Its interpretation can be particularly challenging when the agar medium is darkly pigmented, making it difficult to visually detect colonies within the wells [46]. Also, this method does not provide a complete dose–response curve but only the activity peak that corresponds to the MIC. Despite these limitations, agar dilution offers a significant advantage since it enables testing of high concentrations of a compound. This feature is particularly valuable when screening complex matrices such as phytocomplexes, where the antimicrobial component may be present at relatively low concentrations and within a phytocomplex [48]. The ability to detect antimicrobial activity in such cases lays the groundwork for further investigation and the potential discovery of new potential therapeutic molecules.

Substances that are assessed by broth dilution are generally also testable by the other two methods; however, the converse is not always true. To be testable via broth microdilution, a substance should dissolve completely in water or DMSO, to yield a solution. This condition is essential, as it directly affects the accuracy of spectrophotometric readings and visual assessments of turbidity, as observed during the testing of extract-loaded SiO_2_ nanocomposites. Conversely, compounds that are testable by disk diffusion are not necessarily suitable for broth microdilution, although they are almost always testable via agar dilution. Disk diffusion, when used as the sole screening method, suffers from two critical limitations. First of all, the substance should diffuse out of the paper disk through its matrix. Then, it should be able to migrate through the agar medium, which may physically hinder diffusion depending on its composition.

A literature search on PubMed shows that among the three methods, agar diffusion is the most used (>6000 results), followed by broth microdilution (>5000), and finally agar dilution (just over 1000). Only about 100 studies were found to use all three methodologies side-by-side. However, as highlighted by our data (Figure 3), relying on a single method or a limited combination of techniques may result in the unintentional exclusion of active compounds. Then, it ultimately leads to the loss of potentially promising molecules during the early stages of antimicrobial screening. This corroborates evidence that diffusion assays underestimate activity for viscous/hydrophobic agents [46,53].

The necessity of evaluating all three AST methods—disk diffusion, broth microdilution, and agar dilution—is well established in clinical settings, where determining the most appropriate technique for each compound and even for each bacterial strain is essential. Leading standardization bodies, CLSI and EUCAST, continuously update their guidelines and breakpoint libraries to support this precision-driven approach [30]. Reller et al. (2009) summarize these evolving practices, emphasizing method selection tailored to compound and pathogen type [48].

Our data show that each method exhibits unique detection capabilities (Table 1, Figure 3) with disk diffusion missing 7/18 active substances, broth dilution failing for 10/18 due to solubility issues, and agar dilution detecting all molecules. This underscores why CLSI/EUCAST mandate method selection tailored to compound–pathogen pairs [30,31]. As Reller et al. (2009) emphasize, clinical AST leverages known pharmacodynamics to optimize methods, whereas novel substance screening requires full methodological triangulation to avoid discarding promising leads [48]. 

**Figure 5:**
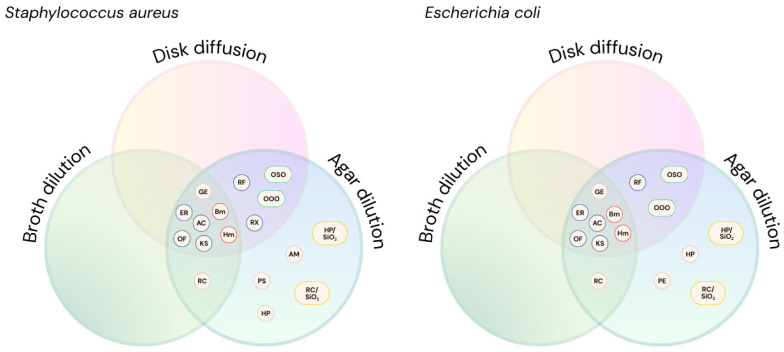
Graphical distribution by Venn diagram of the results obtained using the three methodologies: disk diffusion, agar dilution, and broth dilution. Compounds are color-coded as follows: synthetic antibiotics (blue), oils (green), ionic liquids (orange), silica-based formulations (yellow), and phytochemicals (pink). AM (*Arnica montana* extract), HP (*Harpagophytum procumbens* extract), RC (*Rosa canina* extract), GE (grapefruit extract), PS (polyphenol Solgar), PE (polyphenol Emma), ER (erythromycin), AC (amoxicillin/clavulanic acid), OF (ofloxacin), KS (kanamycin sulfate), RX (rifaximin), RF (rifampicin), Bm (BmimPF_6_), Hm (HmimTFS), OOO (ozonated olive oil), OSO (ozonated sunflower oil), HP/SiO_2_ (*Harpagophytum procumbens*/SiO_2_), RC/SiO_2_ (*Rosa canina*/SiO_2_).

In clinical microbiology, MIC assays are informed by known compound activity, guiding the selection of the most accurate method. In contrast, research contexts—especially when screening novel molecules or, in some cases, phytocomplexes—require comprehensive testing using all three AST methodologies. As shown in Table 2 and Figure 5, each method exhibits varying sensitivity and may detect antimicrobial activity that others miss. Providing a more comprehensive framework for AST by methodological triangulation, our study can help streamline the drug development pipeline, reduce false negatives in early screening, and ultimately accelerate the identification and approval of new therapies to tackle the growing threat of antimicrobial resistance. However, practical challenges arise when testing non-standard materials (e.g., antimicrobial-enhanced face masks, orthopedic alloy samples, or coated biomaterials). In such cases, it may become impossible to apply standard protocols for all three methods. Researchers are often compelled to select a single method, adapting existing ISO procedures or developing creative protocol modifications to assess the compound’s potential under these constraints. For instance, specialized AST adaptations are required when testing surface-bound antimicrobial coatings. Thus, while the ideal workflow includes testing with disk diffusion, broth microdilution, and agar dilution, pragmatic considerations often necessitate method selection or innovation, particularly when working with complex matrices or engineered materials.

Despite the comprehensive comparison of AST methods presented in this study, our analysis was limited to a select group of substances and two bacterial species, which may not fully represent the diversity of chemical structures or microbial targets encountered in clinical and research settings. Future research should expand the panel of compounds and microorganisms, including a broader range of clinically relevant pathogens and resistance phenotypes.

## 4. Materials and Methods

### 4.1. Materials

*Arnica Montana* E.S. 0.1% extract (AM), *Harpagophytum procumbens* extract (HP) and *Rosa canina* L. extract (RC) were bought by Galeno SRL (Carmignano, Italy). Fumed Silica (99.8%) was purchased from Sigma Aldrich (St. Louis, MA, USA). Natural extract/silica nanocomposites were prepared by mechanosynthesis. Polyphenols (PS) were extracted from capsules of Grape Seed Extract of Solgar Inc. (Solgar Inc. 500 Willow Tree Road, Leonia, NJ 07605, USA). Capsules contain 90% of polyphenols and 10% of microcrystalline cellulose, vegetable cellulose, vegetable magnesium stearate and silica. Polyphenols (PE) were extracted from grape powder of Casa Emma (Casa Emma, Società Agricola S.S., S.P. di Castellina in Chianti n.3/5/7, 50028 Barberino Tavernelle—FI, Italy). The product is a flour obtained through a process of drying and grinding grape pomace at low temperature. Pure antibiotics were purchased from Fluka (erythromycin, ER), Sigma (ofloxacin 1 g, OF and rifampicin 100 mg, RF), while commercial antibiotics formulations were purchased from Ratiopharm Italia (amoxicillin/clavulanic acid 875 mg + 125 mg, AC) and Alfasigma (rifaximin 200 mg, RX). Grapefruit extract (GE, Chemie research MFG Co. Inc./P50 Inc., Casselberry, FL, USA). 1-Butyl-3-methylimidazolium hexa-fluorophosphate (BmimPF_6_), >98% was purchased from Merck while 1-Decyl-3-methylimidazolium bis (trifluoromethylsulfonyl)imide (HmimTFS), >98% was purchased from IoLiTec (Ionic Liquid Technologies GmbH, Heilbronn, Germany). Ozonated olive oil (Pure Oil 100%) and ozonated sunflower oil (Pure Sun 100%) were kindly provided by OS Srl (Pesaro, Italy) [31]. The physico-chemical characteristics of the potential antimicrobials tested in this study are reported as material cards in the Supplementary Materials, and the concentrations of the stock solution are reported in Table S1. Dimethyl sulfoxide (DMSO; Sigma Aldrich, Saint Louis, MO, USA).

#### Microbial Strains and Culture Conditions

Bacterial strains were obtained from the American Type Culture Collection (ATCC). *Staphylococcus aureus* (ATCC 6538) and *Escherichia coli* BL21 (DE3) omp8, characterized by the genotype ΔlamB ompF::Tn5 ΔompA ΔompC, were cultured in Tryptic Soy Broth (TSB) and Luria–Bertani Lennox Broth (LB), respectively. All culture media were purchased from Condalab Italia. Stock cultures were maintained at −80 °C in the appropriate broth supplemented with 20% glycerol. For each strain, a preliminary growth curve was performed to determine a standardized inoculum concentration (5 × 10^7^ CFU/mL for all bacteria), aiming to minimize variation in experimental conditions and ensure reproducibility of the results. During the experiments, bacterial cultures were incubated at 37 °C with shaking at 200 rpm for 24 h.

### 4.2. Methods

#### 4.2.1. Mechanosynthesis of Natural Extract/SiO_2_ Nanocomposites

Each extract was grinded with fumed silica in a planetary mill apparatus, Fritsch GmbH, Pulverisette 5 (60 min milling at 100 rpm). Composition of the starting mixture was selected to obtain a dry extract content in the nanocomposite equal to 10 wt%.

Mechanochemistry offers an opportunity to overcome the limits of solution-based harsh conditions, because it uses a trace amount or no solvents. Moreover, it is an easy, cost-efficient and fast synthesis method, in perfect agreement with the Green Chemistry principles [55,56,57,58].

#### 4.2.2. Extraction Procedure

Approximately 1 g of the capsule content was extracted by using an ultrasound-assisted solvent extractor. A 1:1 water/ethanol solution (15 mL) was added to the solid sample and sonication was performed directly on the mixture using a sonicator dip probe, for 3 min, with 3 s pulses of and 1 s pauses. The resulting mixture was centrifuged to recover the red solution, while the solid residue was discarded. The solution was then dried in a 35 °C forced-air oven to remove the alcoholic fumes (flammability hazard). The red precipitate was recovered with a yield higher than 90% and 10% with respect to the starting materials, for PS and PE, respectively. The use of an ultrasound-assisted extractor enables a greener approach compared to conventional solvent-extraction techniques such as ultrasonic baths or Soxhlet extraction. It also represents a cost-effective alternative to supercritical CO_2_ extraction. Using this equipment, 1 g of sample can be processed with only 15 mL of solvent. Since the ultrasound is generated in situ, sonication times are typically an order of magnitude shorter than those required by ultrasonic baths [45,59,60,61,62]. This translates into savings in solvent consumption, energy use, and noise pollution.

#### 4.2.3. Disk Diffusion

All compounds were tested using the agar disk diffusion method as described above. For each bacterial strain, 15 mL of Mueller Hinton (MH) agar medium, maintained at 55 °C, was poured into 90 mm Petri dishes. After solidification, 650 μL of the standardized bacterial suspension (5 × 10^7^ CFU/mL) was inoculated onto the agar surface using a sterile L-shaped spreader. Sterile blank paper disks (5 mm in diameter) were impregnated with 10 μL of each compound dilution and placed on the surface of the inoculated agar. Plates were incubated at 37 °C for 24 h after which they were inspected and photographed. Antimicrobial activity was assessed by the presence of inhibition halos surrounding the disks.

#### 4.2.4. Broth Dilution

Minimum inhibitory concentration (MIC) and minimum bactericidal concentration (MBC) were determined using the broth microdilution method in sterile 96-well plates (Thermo Fisher Scientific, Norristown, PA, USA), following the Clinical and Laboratory Standards Institute (CLSI) guidelines. Each well was filled with 200 μL of standardized microbial suspension and varying concentrations of the tested compounds. Untreated microbial cultures served as the negative control. Plates were incubated at 37 °C for 24 h, and bacterial growth was assessed by measuring optical density at 600 nm (OD_600_) using a microplate reader (Tecan Infinite 200, Männedorf, Switzerland). To determine the MBC, 2 μL from each well was transferred to a new 96-well plate containing 200 μL of fresh medium per well. Following a further 24 h incubation at 37 °C, OD_600_ readings were taken with the same microplate reader to identify the lowest compound concentration that resulted more than 80% compared to the control group growth, and its optical density measure does not significantly differ from that of the original inoculum (baseline at 0 h), as calculated by one-way ANOVA followed by Fisher’s LSD test, setting the significance at *p* < 0.01, performed using Prism 9 (GraphPad Software, San Diego, CA, USA).

#### 4.2.5. Agar Dilution

Serial dilutions of each compound were prepared directly in molten Mueller Hinton agar (MHA) maintained at 55 °C, following prior evaluation confirming that this procedure did not alter the chemical properties of the tested substances. Each compound was incorporated into the agar. Subsequently, 500 μL of the resulting agar–compound mixtures were dispensed into the wells of sterile 24-well plates. Once the agar had solidified, 10 μL of standardized microbial suspension (5 × 10^7^ CFU/mL) was inoculated onto the surface of each well. The plates were incubated at 37 °C for 24 h. After incubation, plates were inspected and photographed, and antimicrobial activity was assessed based on the absence of visible microbial growth. Some wells containing untreated bacterial cultures served as the control group.

#### 4.2.6. Literature Search

The literature search was conducted using the PubMed database, applying the following search strategies: (i) for disk diffusion (antimicrobial) AND ((Kirby Bauer) OR (disk diffusion) OR (disk diffusion) OR (agar diffusion)); (ii) for broth dilution (antimicrobial) AND ((broth dilution) OR (microdilution)); (iii) for agar dilution (antimicrobial) AND (agar dilution). For all the three methodology (antimicrobial) AND ((broth dilution) OR (microdilution)) AND ((Kirby Bauer) OR (disk diffusion) OR (disk diffusion) OR (agar diffusion)) AND (agar dilution).

#### 4.2.7. Statistical Analysis

Data analysis was carried out with the software GraphPad Prism v10 (GraphPad Software, San Diego, CA, USA) for macOS [GraphPad Software. GraphPad Prism. Available online: www.graphpad.com (accessed on 4 August 2025)]. All data were expressed as the means ± standard errors from at least three independent experiments in triplicate and analyzed by the unpaired Student’s *t*-test, Mann–Whitney U test, or 2-way analysis of variance (ANOVA) followed by Fisher’s LSD test. Wilcoxon matched-pairs signed rank test and non-parametric Spearman correlation analysis were also used where indicated. Data were considered significant when *p* < 0.05.

## 5. Conclusions

Method-dependent discrepancies in AST are likely not artifacts but reflections of the complex interactions between compound, medium and pathogen. While agar dilution offers broad detection capability, broth microdilution provides quantitative results, and disk diffusion enables rapid screening, reliance on any individual method risks false negatives. Overall, these results demonstrate that relying on a single AST method in preclinical research may lead to an under- or over-estimation of the antimicrobial potential, particularly for complex natural extracts or novel formulations for which methodological triangulation is crucial. Beyond innovative assay development, a multiparametric approach provides a more comprehensive and accurate assessment of the antimicrobial potential, overcoming the individual methodological limitations.

## Figures and Tables

**Table 1:** Minimum inhibitory concentration (MIC) values of the tested substance against *S. aureus* and *E. coli*, determined by agar dilution, broth dilution, and disk diffusion methods. Data are reported as mean ± SD of concentration (mg/mL) from at least three individual experiments.

Substance		*S. aureus*			*E. coli*		
		Agar Dilution	Broth Dilution	Disk Diffusion	Agar Dilution	Broth Dilution	Disk Diffusion
*Arnica montana* extract	AM	200.00 ± 52.11	>200	>200	>200	>200	>200
*Harpagophytum procumbens* extract	HP	8.33 ± 3.61	>200	>200	4.16 ± 1.81	>200	>200
*Rosa canina* extract	RC	8.33 ± 3.61	6.25 ± 2.18	>200	10.42 ± 3.61	12.5 ± 4.37	>200
Grapefruit extract	GE	0.25 ± 0.08	0.50 ± 0.16	2.50 ± 0.87	2.00 ± 0.07	0.67 ± 0.29	150.00 ± 52.53
Polyphenols Solgar extract	PS	4.65 ± 1.62	>9.3	>186	>9.3	>9.3	>186
Polyphenols EMMA extract	PE	>1.6	>1.6	>32	1.60 ± 0.56	>1.6	>32
Erythromycin	ER	0.0008 ± 0.0003	0.008 ± 0.0028	0.0038 ± 0.0018	0.086 ± 0.037	0.77 ± 0.36	1.28 ± 0.44
Amoxicillin/clavulanic acid	AC	0.0005 ± 0.0002	0.0067 ± 0.0023	0.25 ± 0.08	0.021 ± 0.009	0.008 ± 0.0028	0.21 ± 0.07
Ofloxacin	OF	0.00042 ± 0.00014	0.016 ± 0.0056	0.0083 ± 0.0029	0.000012 ± 0.000006	0.00003 ± 0.00001	0.00013 ± 0.00005
Kanamycin sulfate	KS	0.004 ± 0.0014	0.19 ± 0.09	0.040 ± 0.014	0.010 ± 0.008	0.043 ± 0.018	0.24 ± 0.11
Rifaximin	RX	0.00006 ± 0.00002	>0.128	0.0006 ± 0.0002	>1.024	>0.128	>20.48
Rifampicin	RF	0.000093 ± 0.000032	>0.128	0.00125 ± 0.00043	0.048 ± 0.023	>0.128	1.92 ± 0.91
1-Butyl-3-methylimidazolium hexa-fluorophosphate	BmimPF_6_	69.00 ± 24.15	69.00 ± 24.15	1380.00 ± 438.00	69.00 ± 24.15	69.00 ± 24.15	1380.00 ± 438.00
1-Decyl-3-methyl imidazolium bis (trifluoromethylsulfonyl)imide	HmimTFS	6.40 ± 2.24	64.00 ± 22.40	128.00 ± 44.80	6.40 ± 2.24	64.00 ± 22.40	128.00 ± 44.80
Ozonated olive oil	OOO	1.25 ± 0.43	>50	208.33 ± 72.17	0.83 ± 0.36	>50	125.00 ± 43.75
Ozonated sunflower oil	OSO	1.25 ± 0.43	>50	25.83 ± 8.95	0.83 ± 0.36	>50	500.00 ± 175.00
*Harpagophytum procumbens* extract/SiO_2_	HP/SiO_2_	125.00 ± 43.75	>125	>125	47.25 ± 21.57	>125	>125
*Rosa canina* extract/SiO_2_	RC/SiO_2_	93.75 ± 44.19	>125	>125	93.75 ± 44.19	>125	>125

**Table 2:** Minimum bactericidal concentration (MBC) values of the tested substances against *S. aureus* and *E. coli* were assessed using the broth dilution method. Data are reported as mean ± SD of concentration (mg/mL) from at least three individual experiments.

Substance		*S. aureus*	*E. coli*
*Arnica montana* extract	AM	>200	>200
*Harpagophytum procumbens* extract	HP	>200	>200
*Rosa canina* extract	RC	12.50 ± 4.37	20.83 ± 7.21
Grapefruit Extract	GE	0.83 ± 0.28	1.00 ± 0.35
Polyphenols Solgar extract	PS	>9.3	>9.3
Polyphenols EMMA extract	PE	>1.6	>1.6
Erythromycin	ER	0.016 ± 0.015	>1.024
Amoxicillin/clavulanic acid	AC	0.008 ± 0.028	0.008 ± 0.028
Ofloxacin	OF	0.016 ± 0.005	0.000125 ± 0.000043
Kanamycin sulfate	KS	0.032 ± 0.011	0.032 ± 0.011
Rifaximin	RX	>1.024	>1.024
Rifampicin	RF	>0.128	>1.024
1-Butyl-3-methylimidazolium hexa-fluorophosphate	BmimPF_6_	69.00 ± 24.15	69.00 ± 24.15
1-Decyl-3-methyl imidazolium bis (trifluoromethylsulfonyl)imide	HmimTFS	64.00 ± 22.4	53.33 ± 18.47
Ozonated olive oil	OOO	13.33 ± 5.77	13.33 ± 4.61
Ozonated sunflower oil	OSO	25.00 ± 8.75	30.00 ± 10.5
*Harpagophytum procumbens* extract/SiO_2_	HP/SiO_2_	>125	>125
*Rosa canina* extract/SiO_2_	RC/SiO_2_	>125	>125

## Data Availability

The original contributions presented in this study are included in the article/Supplementary Materials. Further inquiries can be directed to the corresponding author(s).

## Supplementary Materials

The following supporting information can be downloaded at: https://www.mdpi.com/article/10.3390/antibiotics14080848/s1. Table S1. Stock solution concentrations (mg/mL) of the tested compounds used in the three methodologies: disk diffusion, agar dilution, and broth dilution. Table S2. Card of *Harpagophytum procumbens* extract/SiO_2_ nanocomposite sample, including preparation method, composition, physical form, and color. Table S3. Card of *Rosa canina*/SiO_2_ nanocomposite sample, including preparation method, composition, physical form, and color. Table S4. Card of *Arnica montana* L., including sample identity, taxonomic family, geographical origin, composition, active ingredients, extraction solvent, plant source, physical form, color, and density. Data from Galeno technical data sheet. Table S5. Card of *Harpagophytum procumbens*, including sample identity, taxonomic family, geographical origin, composition, active ingredients, extraction solvent, plant source, physical form, color, and density. Data from Galeno technical data sheet. Table S6. Card of *Rosa canina*, including sample identity, taxonomic family, geographical origin, composition, active ingredients, extraction solvent, plant source, physical form, color, and density. Data from Galeno technical data sheet. Table S7. Card of Grapefruit extract, including sample identity, taxonomic family, composition, solubility, color, specific gravity and pH. Data from Galeno technical data sheet. Table S8. Card of 1-Butyl-3-methylimidazolium hexa-fluorophosphate, including physical form, color, molecular weight, density, viscosity, and solubility. Data from Merck technical data sheet. Table S9. Card of 1-Decyl-3 methylimidazolium bis (trifluoromethylsulfonyl)imide, including physical form, color, molecular weight, density, viscosity, and solubility. Data from IoLiTec technical data sheet. Table S10. Card of ozonized olive oil, including composition, physical form, color, density, viscosity, and solubility. Data from OS Srl technical data sheet. Table S11. Card of ozonized sunflower oil, including composition, physical form, color, density, viscosity, and solubility. Data from OS Srl technical data sheet. Table S12. Card of PS extract, including sample identity, composition, active ingredients, extraction solvent, extract form, and color. Table S13. Card of PE extract, including sample identity, composition, active ingredients, extraction solvent, extract form, and color.

## Abbreviations

The following abbreviations are used in this manuscript:

AC	Amoxicillin/clavulanic acid
AM	*Arnica montana* extract
AMR	Antimicrobial resistance
AST	Antimicrobial susceptibility testing
BmimPF_6_	1-Butyl-3-methylimidazolium hexa-fluorophosphate
CLSI	Clinical and Laboratory Standards Institute
ECOFFs	Clinical breakpoints and epidemiological cut-off values
ER	Erythromycin
EUCAST	European Committee on Antimicrobial Susceptibility Testing
GE	Grapefruit Extract
HP	*Harpagophytum procumbens* extract
HP/SiO_2_	*Harpagophytum procumbens* extract/SiO_2_
HmimTFS	1-Decyl-3-methylimidazolium bis (trifluoromethylsulfonyl)imide
IHME	Institute for Health Metrics and Evaluation
KS	Kanamycin sulfate
MBC	Minimum Bactericidal Concentration
MHA	Muller Hinton Agar
MIC	Minimum Inhibitory Concentration
OF	Ofloxacin
OOO	Ozonated olive oil
OSO	Ozonated sunflower oil
PE	Polyphenol Emma extract
PS	Polyphenol Solgar extract
RC	*Rosa canina* extract
RC/SiO_2_	*Rosa canina* extract/SiO_2_
RF	Rifampicin
RX	Rifaximin
