# Supplementary material for: Beyond One-Size-Fits-All: Addressing Methodological Constraints in Novel Antimicrobials Discovery
Source: Antibiotics (Basel). 2025 Aug 21;14(8):848. doi: 10.3390/antibiotics14080848 (PMC12382792; doi:10.3390/antibiotics14080848)
Supplement: Supplementary file 1 [file antibiotics-14-00848-s001.zip › antibiotics-3783240-supplementary.pdf]

antibiotics

Supplementary

Beyond one-size-fits-all: Addressing Methodological Constraints in Novel Antimicrobials Discovery

Silvia Puxeddu [1†], Serena Canton [1†], Alessandra Scano [2,3,*], Ilenia Delogu [1], Andrea Pibiri [1], Cristiana Cabriolu [2], Sarah Vascellari [1], Francesca Pettinau [2,4], Tiziana Pivetta [2], Guido Ennas [2,3], Aldo Manzin [1] and Fabrizio Angius [1,*]

[1] Department of Biomedical Sciences, Section of Microbiology and Virology, University of Cagliari, Cagliari, Italy; s.puxeddu1@studenti.unica.it (S.P.); serena.canton@unica.it (S.C.); ilenia.delogu@unica.it (I.D.); a.pibiri4@studenti.unica.it (A.P.); svascellari@unica.it (S.V.); aldomanzin@unica.it (A.M.)
[2] Department of Chemical and Geological Sciences, University of Cagliari, Cagliari, Italy; cristiana.cabriolu@unica.it (C.C.); tpivetta@unica.it (T.P.); ennas@unica.it (G.E.)
[3] Research Unit of the National Consortium of Materials Science and Technology (INSTM), University of Cagliari, Cagliari, Italy
[4] Institute of Translational Pharmacology, National Research Council, Pula (CA), Italy; francesca.pettinau@ift.cnr.it (F.P.)
* Correspondence: fangius@unica.it (F.A.); alessandra.scano@unica.it (A.S.)
† These authors contributed equally to this work

Supplementary

 https://doi.org/10.3390/antibiotics14080848

Table S1. Stock solution concentrations (mg/mL) of the tested substances used in the three methodologies: disk diffusion, agar dilution, and broth dilution.

**Substance**		**Stock solution mg/mL (solvent)**		
		**Agar dilution**	**Broth dilution**	**Disk diffusion**
Arnica montana extract	AM	200 (MHA)	200 (Broth)	200 (water)
Harpagophytum procumbens extract	HP	200 (MHA)	200 (Broth)	200 (water)
Rosa canina extract	RC	200 (MHA)	200 (Broth)	200 (water)
Grapefruit Extract	GE	1200 (MHA)	1200 (water)	1200 (water)
Polyphenols Solgar extract	PS	186 (MHA)	186 (50% ethanol)	186 (50% ethanol)
Polyphenols EMMA extract	PE	31.9 (MHA)	31.9 (50% ethanol)	31.9 (50% ethanol)
Erythromycin	ER	20.48 (MHA)	20.48 (water)	20.48 (water)
Amoxicillin/clavulanic acid	AC	2.56 (MHA)	2.56 (water)	2.56 (water)
Ofloxacin	OF	2.56 (MHA)	2.56 (water)	2.56 (water)
Kanamycin sulfate	KS	5 (MHA)	5 (water)	5 (water)
Rifaximin	RX	20.48 (MHA)	20.48 (water)	20.48 (water)
Rifampicin	RF	20.48 (MHA)	20.48 (water)	20.48 (water)
1-Butyl-3-methyllimidazolium hexa-fluorophosphate	$BmimPF_6$	1380 (MHA)	1380 (DMSO)	1380 (DMSO)
1-Decyl-3 methyl imidazoliumbis (trifluoromethylsulfonyl)imide	HmimTFS	1280 (MHA)	1280 (DMSO)	1280 (DMSO)
Ozonated olive oil	OOO	1000 (MHA)	1000 (water)	1000 (DMSO)
Ozonated sunflower oil	OSO	1000 (MHA)	1000 (water)	1000 (DMSO)
Harpagophytum procumbens extract /SiO_2	HE/SiO_2	125 (MHA)	125 (Broth)	125 (water)
Rosa canina extract/SiO_2	RC/SiO_2	125 (MHA)	125 (Broth)	125 (water)

Table S2. Card of *Arnica montana L.*, including sample identity, taxonomic family, geographical origin, composition, active ingredients, extraction solvent, plant source, physical form, color, and density. Data from Galeno technical data sheet.

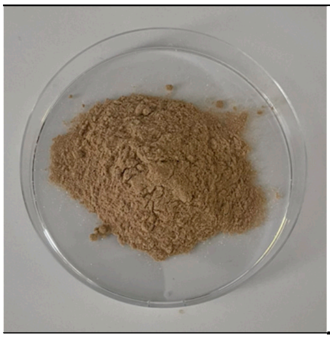	***Arnica montana L.***
**Sample**	*Arnica montana* E.S. 0.1%
**Family**	Asteraceae
**Origin**	East Europe, Mexico
**Composition**	*Arnica montana* extract, corn maltodextrin
**Active ingredients**	Sesquiterpene lactones, essential oil, triterpen, flavonoids and cumarine
**Extraction solvent**	Ethanol/water 50:50
**Plant source**	Flower
**Extract form**	Powder
**Color**	Brown
**Density**	0.4 - 0.6 g/mL

Table S3. Card of *Harpagophytum procumbens*, including sample identity, taxonomic family, geographical origin, composition, active ingredients, extraction solvent, plant source, physical form, color, and density. Data from Galeno technical data sheet.

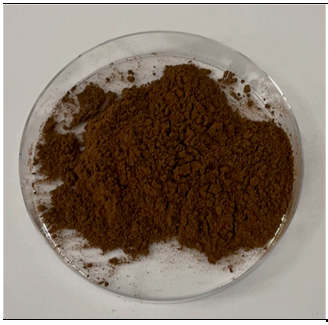	***Harpagophytum procumbens***
**Sample**	*Harpagophytum procumbens* E.S. 20% harpagoside
**Family**	Pedaliaceae
**Origin**	China
**Composition**	*Harpagophytum procumbens* extract, corn maltodextrin
**Active ingredients**	Indol glucosides triterpens, flavonoids, phenolic acids, polysaccharides
**Extraction solvent**	Ethanol/water
**Plant source**	Root
**Extract form**	Powder
**Color**	Yellow-brown
**Density**	0.3 - 0.7 g/mL

Table S4. Card of *Rosa canina*, including sample identity, taxonomic family, geographical origin, composition, active ingredients, extraction solvent, plant source, physical form, color, and density. Data from Galeno technical data sheet.

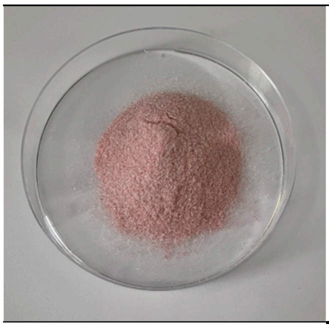	***Rosa canina L.***
**Sample**	*Rosa canina* dry extract, 70% Vitamin C
**Family**	Rosaceae
**Origin**	Europe
**Composition**	*Rosa canina* extract, vitamin C, corn maltodextrin
**Active ingredients**	Vitamin C, carotenoids, organic acids, flavonoids and polyphenols
**Extraction solvent**	Water
**Plant source**	Pseudocarp
**Extract form**	Powder
**Color**	Pink
**Density**	0.5 g/mL

Table S5. Card of Grapefruit extract, including sample identity, taxonomic family, composition, solubility, color, specific gravity and pH. Data from Galeno technical data sheet.

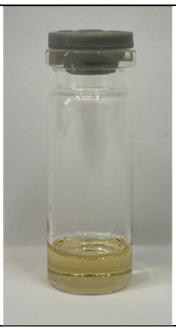	**Grapefruit extract**
**Sample**	*Citrus grandis*
**Family**	Rutaceae
**Origin**	Commercial
**Composition**	*Citrus grandis* 45%, glycerin 35%, ascorbic acid 18%, moisture 2%
**Solubility**	Water, alcohol and organic solvents
**Color**	Yellow
**Specific gravity**	1.10 - 1.30 at 20°C
**pH**	1.5 - 3.0 at 25°C

Table S6. Card of Polyphenol Solgar extract, including sample identity, composition, active ingredients, extraction solvent, extract form, and color.

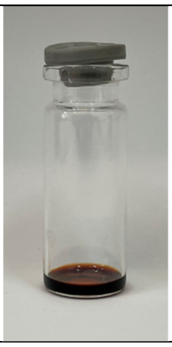	**Polyphenol Solgar extract**
**Sample**	Grape powder Solgar
**Origin**	Commercial
**Composition**	Polyphenols and other ingredients
**Active ingredients**	Polyphenols
**Extraction solvent**	Ethanol/water 50:50
**Extract form**	Brilliant solid then dispersed in pure ethanol
**Color**	Red

Table S7. Card of Polyphenol Emma extract, including sample identity, composition, active ingredients, extraction solvent, extract form, and color.

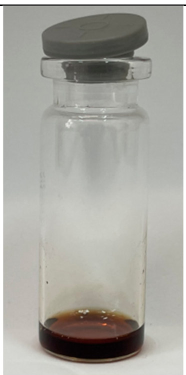	**Polyphenol Emma extract**
**Sample**	Grape powder Casa Emma
**Origin**	Commercial
**Composition**	Polyphenols and other ingredients
**Active ingredients**	Polyphenols
**Extraction solvent**	Ethanol/water 50:50
**Extract form**	Brilliant solid then dispersed in pure ethanol
**Color**	Red

Table S8. Card of 1-Butyl-3-methylimidazolium hexa-fluorophosphate, including physical form, color, molecular weight, density, viscosity, and solubility. Data from Merck technical data sheet.

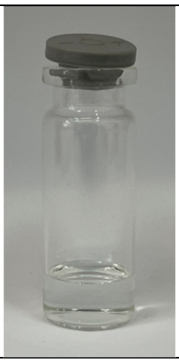	**1-Butyl-3-methylimidazolium hexa-fluorophosphate**
**Acronym**	$BmimPF_6$
**Form**	Viscous Liquid
**Color**	Colorless to light brown-yellow
**Molecular weight**	284.18 g/mol
**Density**	1.38 g/mL at 20 °C
**Viscosity**	310 mPa * s at 25° C
**Solubility**	Soluble in CH_2Cl_2, $CHCl_3$, and EtOAc; immiscible with water and many nonpolar organic solvents such as diethyl ether and hexane

Table S9. Card of 1-Decyl-3 methylimidazoliumbis (trifluoromethylsulfonyl)imide, including physical form, color, molecular weight, density, viscosity, and solubility. Data from IoLiTec technical data sheet.

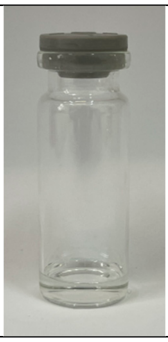	**1-Decyl-3 methylimidazoliumbis (trifluoromethylsulfonyl)imide**
**Acronym**	HmimTFS
**Form**	Viscous Liquid
**Color**	Colorless to yellow
**Molecular weight**	503.53 g/mol
**Density**	1.28 g/mL at 20 °C
**Viscosity**	137 mPa * s at 24 °C
**Solubility**	Miscible in acetone, isopropanol and toluene; immiscible with water and hexane

Table S10. Card of ozonized olive oil, including composition, physical form, color, density, viscosity, and solubility. Data from OS Srl technical data sheet.

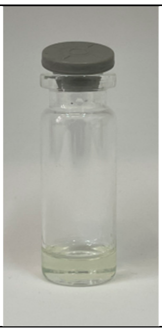	**Ozonized Olive Oil**
**Composition**	Ozonized *Oleaeuropea* (olive) fruit with Ozone
**Form**	Translucent, slightly opalescent oily liquid
**Color**	Light straw yellow
**Density**	0.900 g/mL at 20 °C
**Viscosity**	86.0 mPa * s at 25° C
**Solubility**	Soluble in glycerin/paraffin; fat-soluble in oils and dispersible in emulsions

Table S11. Card of ozonized sunflower oil, including composition, physical form, color, density, viscosity, and solubility. Data from OS Srl technical data sheet.

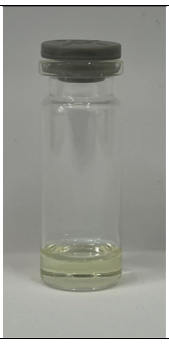	**Ozonized Sunflower Oil**
**Composition**	Ozonized Sunflower Seed Oil - *Heliantus annuus* (sunflower) seed oil with Ozone
**Form**	Translucent, slightly opalescent oily liquid
**Color**	Straw yellow
**Density**	0.990 g/mL at 20 °C
**Viscosity**	180.0 mPa * s at 25° C
**Solubility**	Soluble in glycerin/paraffin; fat-soluble in oils and dispersible in emulsions

Table S12. Card of *Harpagophytum procumbens* extract / SiO_2 nanocomposite sample, including preparation method, composition, physical form, and color.

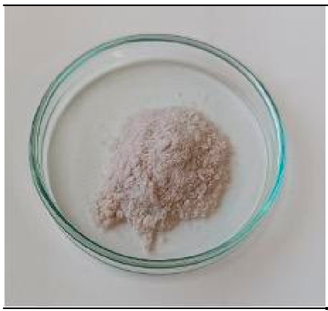	***Harpagophytum procumbens* extract / SiO_2 nanocomposite**
**Composition**	*Harpagophytum procumbens* extract (10 wt%) and fumed silica
**Form**	Powder
**Color**	Light brown
**Preparation method**	Mechanosynthesis (planetary mill apparatus Fritsch GmbH Pulverisette 5; 60 min grinding; 100 rpm speed)

Table S13. Card of *Rosa canina* / SiO_2 nanocomposite sample, including preparation method, composition, physical form, and color.

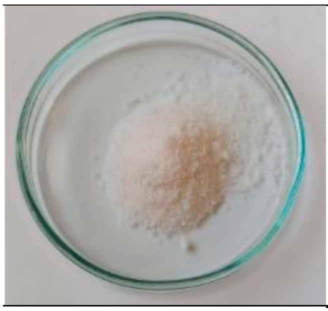	***Rosa canina* / SiO_2 nanocomposite**
**Composition**	*Rosa canina* extract (10 wt%) and fumed silica
**Form**	Powder
**Color**	Light pink
**Preparation method**	Planetary mill apparatus Fritsch GmbH Pulverisette 5; 60 min grinding; 100 rpm speed
